# Blindness among Patients with Type II Diabetes Mellitus Presenting to the Outpatient Department of Ophthalmology of a Tertiary Care Centre: A Descriptive Cross-sectional Study

**DOI:** 10.31729/jnma.7702

**Published:** 2022-10-31

**Authors:** Pooja Shrestha, Raju Kaiti, Ranjila Shyangbo

**Affiliations:** 1 Department of Ophthalmology, Kathmandu University School of Medical Sciences, Dhulikhel, Kavre, Nepal; 2 Department of Optometry, Nepal Eye Hospital, Tripureshwor, Kathmandu, Nepal; 3 Department of Optometry, National Academy of Medical Sciences, Mahaboudha, Kathmandu, Nepal

**Keywords:** *blindness*, *diabetic retinopathy*, *prevalence*

## Abstract

**Introduction:**

Diabetic retinopathy is a major microvascular complication of diabetes, and may progress to sight-threatening stages causing blindness with a consequent decrease in their quality of life. This study aimed to find out the prevalence of blindness among patients with type II diabetes mellitus attending the Outpatient Department of Ophthalmology of a tertiary care hospital.

**Methods:**

A descriptive cross-sectional study was conducted among patients with type II diabetes mellitus presenting to the Outpatient Department of Ophthalmology of a tertiary care centre from 2 August 2021 to 30 June 2022 after receiving ethical approval from the Institutional Review Committee (Reference number: 74/2021). Diabetic patients underwent detailed eye examination including vision, slit lamp biomicroscopy examination, and fundus evaluation with full pupil dilation. Convenience sampling method was used. Point estimate and 95% Confidence Interval were calculated.

**Results:**

Among 449 type II diabetic patients, blindness was seen in 17 (3.79%) (2.02-5.56, 95% Confidence Interval) patients. Among them, 1 (5.88%) had severe non-proliferative diabetic retinopathy, 3 (17.65%) had proliferative diabetic retinopathy and 8 (47.06%) had severe diabetic macular oedema.

**Conclusions:**

The prevalence of blindness among patients with type II diabetes mellitus was less than in other studies conducted in similar settings. Screening and timely management of diabetic retinopathy could reduce the prevalence of blindness due to diabetic retinopathy.

## INTRODUCTION

Diabetic retinopathy (DR), a microvascular complication of both type I and type II diabetes, may progress to sight-threatening stages causing visual impairment and blindness if not well controlled. World Health Organization (WHO) has estimated that diabetic retinopathy is responsible for 4.8% of the 37 million cases of blindness throughout the world.^1^

According to WHO, there were 436,000 diabetic cases in Nepal in the year 2000 but by the year 2030, it will increase to 1,328,000 cases.^2^ So, with this increasing trend of diabetes in Nepal, complications due to Diabetes mellitus (DM) are likely to increase including visual impairment and blindness but there is no existing data regarding this situation.

This study aimed to estimate the prevalence of blindness among patients with type II diabetes mellitus attending the Outpatient Department of Ophthalmology of a tertiary care centre.

## METHODS

A descriptive cross-sectional study was conducted among patients with type II diabetes mellitus presenting to the Outpatient Department of Ophthalmology of Dhulikhel Hospital from 2 August 2021 to 30 June 2022 after obtaining ethical approval from the Institutional Review Committee (Reference number: 74/2021). Informed written consent was obtained from all the participants of this study. All the type II diabetic patients, of any age and gender, presented within the study period were included in the study. Patients with mature cataracts, patients having anterior segment and other posterior segment diseases were excluded from the study. Convenience sampling was done. The sample size was calculated using the following formula.


$$\begin{array}{ll} \text{n} & ={\text{Z}}^{2}\times \frac{\text{p}\times \text{q}}{{\text{e}}^{\text{2}}}\\ & =1.96^{2}\times \frac{0.50\times 0.50}{0.05^{2}}\\ & =385 \end{array}$$

Where,

n= minimum required sample size
Z= 1.96 at 95% Confidence Interval (CI)
p= prevalence taken as 50% for maximum sample size
q= 1-p
e= margin of error of, 5%

The minimum sample size calculated was 385. However, 449 patients were included in the study.

Demographicdetailsand information regarding diabetes mellitus and treatment received were obtained from each participant. All the patients underwent eye examinations: distant visual acuity and near vision assessment followed by retinoscopy. The best corrected visual acuity (BCVA) was noted for each participant. Then, a slit lamp biomicroscopy examination was performed to detect any anterior segment and posterior segment abnormalities. They underwent full dilatation of the pupil with a combination of 0.8% tropicamide and 5% phenylephrine eye drop followed by fundus evaluation then status and grade of diabetic retinopathy were recorded. A fundus photograph was taken and recorded with diabetic retinopathy whenever feasible. Classification of diabetic retinopathy was done according to the International Classification of Diabetic Retinopathy Scale.^3^ According to the best corrected visual acuity of the patients, blindness was graded according to the WHO classification of blindness.^4^

Data were collected and entered in Microsoft Excel 2011 and analysed in IBM SPSS Statistics 11.5. Point estimate and 95% CI were calculated.

## RESULTS

Among 449 patients with type II diabetes mellitus, blindness was seen in 17 (3.79%) (2.02-5.56, 95% CI) patients. Among them, 1 (5.88%) had severe nonproliferative diabetic retinopathy (NPDR), 3 (17.65%) had proliferative diabetic retinopathy (PDR) and 8 (47.06%) had severe diabetic macular oedema (DMO) (Table 1).

**Table 1:** Grading of diabetic retinopathy in patients with blindness (n= 17).

Grade of diabetic retinopathy	n (%)
Mild NPDR	-
Moderate NPDR	4 (23.53)
Severe NPDR	1 (5.88)
PDR	3 (17.65)
Mild DMO	-
Moderate DMO	1 (5.88)
Severe DMO	8 (47.06)

Blindness in either eye was found in 10 (58.82%) male and seven (41.18%) female. The mean age of the patients having blindness was 61.94±15.24 years. Among 17 patients, 6 (35.29%) patients were of the age group 70 years or above whereas only 2 (11.76) patients were of the age group of 40 years or less. The mean duration of diabetes mellitus in the 17 patients was 14.37±8.02 years. In only 7 (41.10%) patients, the duration of DM was 11 to 15 years whereas only 4 (23.53%) patients had DM duration of 21 years or more. No patients within 1 year or less duration of DM were blind. A total of 9 (52.94%) patients were only on oral hypoglycemic agents (OHA) and 8 (47.06%) patients were on both OHA and insulin (Table 2).

**Table 2:** Socio-demographic characteristics (n= 17).

Patient parameters	n (%)
**Sex**	
Male	10 (58.82)
Female	7 (41.18)
**Age group (in years)**	
≤40	2 (11.76)
41 to 50	2 (11.76)
51 to 60	3 (17.65)
61 to 70	4 (23.53)
≥70	6 (35.29)
**Duration of DM (in years)**	
≤1	-
2 to 5	2 (11.76)
6 to 10	2 (11.76)
11 to 15	7 (41.18)
16 to 20	2 (11.76)
≥21	4 (23.53)
**Treatment for DM**	
OHA	9 (52.94)
Insulin	-
Both OHA and insulin	8 (47.06)

## DISCUSSION

In this study, the prevalence of blindness among the type II diabetes patients was 17 (3.79%) which is comparable to study conducted in Nigeria as both were hospital based study with similar study population.^5^

The prevalence of blindness varies widely and has been reported almost zero in most parts of Africa, to three to seven percent in South-East Asia and Western Pacific to high as 15-17% in America and Europe.^6^ The prevalence of blindness in our study is lower than in Bhaktapur Retina Study where diabetic retinopathy was responsible for 5.74% of bilateral low vision and 7.89% of unilateral blindness in the patients and other studies done in Ghana, Nigeria and Jordan due to the different study criterias, settings and study population.^7-10^ Our prevalence was even more lower when compared to a study conducted in Northeast China where 29.6% of blindness was observed in patients with diabetic retinopathy while the leading cause of blindness was due to proliferative diabetic retinopathy in 45.4% of study cohort and another study conducted in Mexico had 29.1% of blindness.^11,12^ Their higher prevalence may be due to the fact that they included diabetic patients with cataract and uncorrected refractive errors also in their inclusion criteria which was exclusion criteria for our study population and maximum number of patients had short duration of diabetes in our study as it is known fact that more than 10 years of diabetes doubles the risk of visual impairment and blindness.^13^

Furthermore, studies done in England, Copenhagen and SN-DREAMS study in Chennai, India, the prevalence of blindness was lower than our study due to different study population and different settings.^14-16^ Globally also, diabetic retinopathy accounts for five percent of all blindness and is the leading cause of blindness in people aged 15 to 64 years in industrialized countries which is higher than our study.^2^ Furthermore, our study was conducted among the known type 2 diabetics only and patients with undiagnosed diabetes might have lead to underestimation of visual impairment and blindness. But increasing awareness about DR and its ocular complications, improved management of diabetes and availability of laser and anti-VEGF treatment are likely to cause reduced prevalence of blindness in the future.

Vision loss in approximately 25% of patients with DR is associated with progression of non proliferative DR to proliferative DR. PDR and DME are both sight-threatening conditions and can result in blindness. Worldwide, an estimated 17 million diabetic people have PDR and without appropriate treatment more than half of the patients with high-risk PDR will be blind within five years. In 1981 Nepal blindness survey by Nepal Netra Jyoti Sangh, 13.9% of blindness was due to diabetic retinopathy, posterior segment and Central nervous system diseases which increased to 17% in 2006 to 2010 blindness survey.^17^

In our study, the prevalence of blindness was observed to be higher in severe diabetic retinopathy. In our study, patients with mild NPDR had no blindness, in patients having blindness, 3 (17.65%) patients had PDR but 8 (47.06%) patients had severe DMO. About 50% of blindness is preventable by early detection and management of PDR and DME which can be done by regular follow up of patients with PDR and DME, laser photocoagulation and intensive blood sugar control.^18^ Similarly, a study conducted in Tunisia also reported that prevalence of blindness was observed to be increased in severe DR where prevalence of blindness increased from 5.3% in mild and moderate NPDR to 18.7% in severe NPDR and progressed to 46.8% in PDR.^13^ In a Jordanian diabetic population also, blindness was significantly associated with severity of DR.^10^ Adequate literature are not available regarding the relation of blindness with severity of diabetic retinopathy in Nepal and could not be explored in this study too. However, primary interventions such as intensive glycemic and blood pressure control can reduce the prevalence of DR, while secondary interventions, such as laser photocoagulation, may prevent further progression of DR and vision loss. Although refractive error correction could improve useful vision significantly in patients with diabetic retinopathy, it cannot eliminate the visual loss.^19^

This study has few limitations. Data were collected from known diabetic cases only so patients with undiagnosed diabetes may have lead to underestimation of prevalence of blindness. This was a single hospital-based crosssectional study so results cannot be applied and interpreted to the general Nepalese population. Optical coherence tomography (OCT) was not used to evaluate macular edema in this study due to its unavailability so mild cases of DME may have been missed. Due to the cross-sectional study design, patients were not followed up so progression of blindness due to progression in DR may have been missed. However, this study provides the baseline data which may be useful for planning interventional strategies like primary prevention, prophylactic treatment with laser or intravitreal injections for management of PDR and DME. Nevertheless, such data estimates are very important for providing baseline data on the basis of which screening and public health programs can be planned to reduce the risk of blindness due to diabetic retinopathy.

## CONCLUSIONS

The prevalence of blindness among type II diabetic patients was lower than in other studies conducted in similar settings. Since our study population had a short duration of diabetes, we need to follow up these cases to assess for increasing severity of blindness so that appropriate interventions can be applied timely.
